# The Copetti Sign in Suspected Renal Colic: Association with Distal Ureteral Stones in a Prospective Pilot Cohort

**DOI:** 10.3390/healthcare14121663

**Published:** 2026-06-11

**Authors:** Carmine Cristiano Di Gioia, Daniele Orso, Alice Alame, Jessica Vella, Michela D’Apolito, Gianmarco Sicuranza, Eli Ollari, Lorenzo Bianchi, Marcello Boccardi

**Affiliations:** 1 Department of Emergency Medicine, Community Hospital of Baggiovara (MO), Azienda Ospedaliero-Universitaria di Modena, 41125 Modena, Italy; digioia.cristiano@aou.mo.it (C.C.D.G.);; 2 Department of Emergency, University Hospital “Santa Maria Della Misericordia”, Azienda Sanitaria Universitaria Friuli Centrale (ASUFC), 33100 Udine, Italy; 3 Department of Urology, University Hospital of Modena and Reggio Emilia, 41125 Modena, Italy

**Keywords:** Copetti sign, renal colic, point-of-care ultrasound, ureteral stones, emergency department

## Abstract

**Highlights:**

**What are the main findings?**

- In this prospective pilot cohort of 44 adults with suspected renal colic, the Copetti sign was identified in 70.5% of patients and was significantly associated with distal ureteral stone location.
- The Copetti sign showed encouraging diagnostic performance for distal ureteral stones, with 88.5% sensitivity, 55.6% specificity, 74.2% positive predictive value, and 76.9% negative predictive value.

**What are the implications of the main findings?**

- The Copetti sign may represent a useful dynamic POCUS marker to complement traditional ultrasound findings in the bedside evaluation of suspected renal colic.
- Its association with conservative management is clinically relevant but exploratory; larger multicenter studies are needed to validate whether it can support imaging decisions, treatment pathways, and patient-centered outcomes.

**Abstract:**

**Background**: Renal colic is a common emergency department (ED) presentation requiring rapid and accurate diagnosis. Point-of-care ultrasound (POCUS) is a fast, radiation-free alternative to CT. The Copetti sign—a rhythmic anteroposterior oscillation of the affected kidney—has been proposed as a dynamic sonographic marker of distal ureteral stones. **Methods**: In this prospective observational study (June–September 2025), 44 adult patients with suspected renal colic were enrolled at a single-center ED in Baggiovara, Modena, Italy. All underwent standardized POCUS to detect the Copetti sign prior to confirmatory imaging. Clinical, laboratory, and sonographic variables were collected. Associations between the Copetti sign, stone location, stone size, hydronephrosis, urinoma, and management strategy were explored. Associations with conservative management were considered exploratory. **Results**: The Copetti sign was identified in 70.5% of patients and was significantly associated with distal ureteral stones (74.2% vs. 25.8%, *p* = 0.005). Copetti-positive patients were more often managed conservatively (77.4% vs. 38.5%, *p* = 0.019), although this likely reflects the underlying clinical-imaging phenotype and local treatment decisions rather than a validated prognostic endpoint. **Conclusions**: In this prospective pilot cohort, the Copetti sign was frequently observed and was strongly associated with distal ureteral stone location. Copetti-positive patients were also more often managed conservatively, although this should be interpreted as an exploratory association reflecting clinical decision-making rather than a validated prognostic endpoint.

## 1. Introduction

Renal colic is a common and challenging emergency department (ED) presentation characterized by severe pain often due to nephrolithiasis [1]. Preventing complications and tailoring management strategies requires rapid and accurate diagnosis. Non-contrast computed tomography (CT) has been traditionally used to detect ureteral stones due to its high sensitivity and specificity [2]. However, CT’s drawbacks—including radiation exposure, high cost, and delays—have prompted interest in alternative imaging approaches [3].

Point-of-care ultrasound (POCUS) has become a valuable diagnostic tool in the ED by allowing rapid bedside evaluation without radiation risks and at lower cost [4]. Ultrasound has some limitations because it relies heavily on indirect signs, such as hydronephrosis, asymmetric ureteral jets, and the twinkling artifact [5]. Due to anatomical and technical limitations, direct visualization of ureteral stones via ultrasound can be challenging [6]. Therefore, there is a growing need for new sonographic markers that enhance diagnostic accuracy. Unlike traditional static ultrasound findings, such as hydronephrosis or direct stone visualization, dynamic sonographic markers are defined by motion-related phenomena observed during real-time scanning and may provide complementary physiologic information.

One such dynamic marker is the Copetti sign, also known as the ‘swinging kidney’ sign, defined as a rhythmic anteroposterior oscillation of the affected kidney along its long axis [7] (Video S1). Early studies indicate that this sonographic phenomenon correlates with distal ureteral stones, particularly in the juxta vesical region, as well as with smaller stone sizes. In addition, its presence suggests conservative management and even spontaneous stone passage [7]. Foundational work by Castelletto et al. noted that the sign was observed in about 70% of patients, significantly associated with stones under 10 mm and favorable prognostic indicators such as reduced hydronephrosis and distal stone location. Integrating this dynamic marker into POCUS protocols may enhance the diagnostic and prognostic roles of ultrasound in the ED.

The primary aim was to assess whether the Copetti sign was associated with distal ureteral stone location among adults with suspected renal colic. Secondary aims were to explore its association with stone size, hydronephrosis, urinoma, and management strategy.

## 2. Materials and Methods

### 2.1. Study Design and Setting

From June to September 2025, a prospective observational study was conducted at a single-center emergency department in Baggiovara, Modena, Italy. The primary analysis examined whether Copetti sign positivity was associated with distal ureteral stone location in patients with suspected renal colic. Institutional Review Board approval was obtained (Prot. AOU 0016637/25; 11 June 2025) and all participants provided written informed consent in accordance with the Declaration of Helsinki. The data collection was in accordance with established observational research guidelines, with standardized data collection, quality assurance measures, and independent verification.

### 2.2. Patient Population

Adult patients (≥18 years) presenting with clinical signs and symptoms consistent with renal colic were consecutively enrolled. The presence of sudden-onset severe flank pain that could radiate to the groin and physical exam findings suggesting ureteral obstruction led to clinical suspicion of renal colic. Exclusion criteria included hemodynamic instability (systolic BP < 90 mm Hg or signs of shock), recent urological interventions (within 30 days), or inability to undergo POCUS evaluation due to body habitus, severe pain, or logistical challenges. Alternative diagnoses, including aortic dissection and non-urological causes of acute abdomen, were ruled out as clinically appropriate. Patient eligibility and reasons for exclusion were documented in a screening log.

### 2.3. Ultrasound Assessment and Data Collection

**Figure 1:**
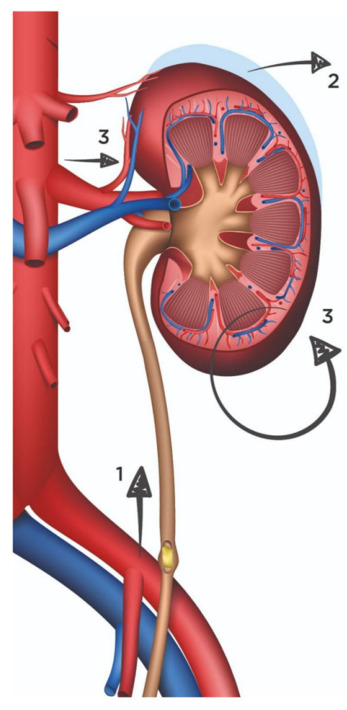
A cross-sectional view of the kidney is used to illustrate a hypothesized mechanism potentially underlying the Copetti sign. Arrow 1 shows that the ureter is obstructed by a distal stone. A rise in pelvic pressure is accompanied by the accumulation of urine in the Gerota’s space (the urinoma is almost invisible). Arrow 3 indicates the oscillating movement of the kidney within Gerota’s fascia caused by aortic pulsation.

All patients enrolled underwent a standardized POCUS exam using a high-resolution ultrasound system with convex and sector transducers for optimal imaging. Experienced ED physicians—trained specifically to identify dynamic renal signs—performed the examinations following a uniform imaging protocol. Both longitudinal and transverse views of the kidneys were acquired. Operators specifically searched for the Copetti sign, defined a priori as a rhythmic, anteroposterior oscillatory movement of the affected kidney along its long axis that could not be solely attributed to respiratory motion and was considered consistent with transmission of aortic pulsatility (Figure 1). The proposed mechanism remains speculative and has not been directly demonstrated.

Data were recorded on a structured case report form and included demographic details, clinical presentation (including pain onset, duration, and character), and laboratory tests (e.g., serum creatinine, urine analysis, inflammatory markers). Stone size and location were recorded on confirmatory non-contrast CT, which was performed in all included patients and served as the reference standard for ureteral stone diagnosis and location. Ancillary sonographic findings, including hydronephrosis and the twinkling artifact, were recorded during the POCUS examination. Management strategies were prospectively recorded, documenting whether patients received conservative treatment (e.g., medical expulsive therapy, analgesia, observation), interventional procedures (e.g., ureteroscopic extraction, percutaneous nephrostomy), or both. Conservative and interventional management were not mutually exclusive, as some patients initially managed conservatively subsequently underwent an intervention. Follow-up data on stone expulsion and clinical outcomes were obtained from subsequent visits or scheduled follow-up.

First, we explored which clinical and imaging variables were associated with the presence of the Copetti sign. Associations between the Copetti sign and management strategy were analyzed as secondary exploratory outcomes. Because treatment was not protocolized, these analyses were interpreted as reflecting local clinical decision-making rather than causal or prognostic effects.

### 2.4. Statistical Analysis

Categorical variables were compared using Fisher’s exact test or chi-square test, as appropriate, and continuous variables using the Kruskal–Wallis test. Associations between clinical or sonographic variables and Copetti sign positivity were summarized using unadjusted odds ratios with 95% confidence intervals. Given the small sample size and sparse cells, these estimates were considered exploratory. The primary analysis focused on the association between Copetti sign positivity and distal ureteral stone location. Secondary analyses explored associations with stone size, hydronephrosis, urinoma, and management strategy. Diagnostic performance for distal ureteral stone location was summarized using sensitivity, specificity, positive predictive value, and negative predictive value. Exact (Clopper–Pearson) 95% confidence intervals were calculated for sensitivity, specificity, positive predictive value, and negative predictive value. Analyses were performed using R software version 4.5.0. Two-sided *p*-values < 0.05 were considered statistically significant.

## 3. Results

A total of 44 patients with complete data were included in the analysis. All included patients underwent confirmatory non-contrast CT imaging. The Copetti sign was identified in 70.5% of cases (31/44). Baseline characteristics are presented in Table 1. There were no statistically significant differences between groups in terms of sex distribution (*p* = 0.845), fever (*p* = 0.071), acute kidney injury (*p* = 0.903), or inflammatory status (*p* = 0.723). However, distal stone location was significantly associated with the presence of the Copetti sign (74.2% vs. 25.8%, *p* = 0.005). Conservative management was attempted more frequently in Copetti-positive patients (77.4% vs. 38.5%, *p* = 0.019). Because conservative and interventional management were not mutually exclusive, these findings should be interpreted as treatment patterns rather than discrete outcome categories. Urinoma was significantly more frequent in Copetti-positive patients (53.3% vs 7.7%, *p* = 0.013; Table 1). Stone size tended to be smaller among Copetti-positive patients, but this difference did not reach statistical significance.

**Figure 2:**
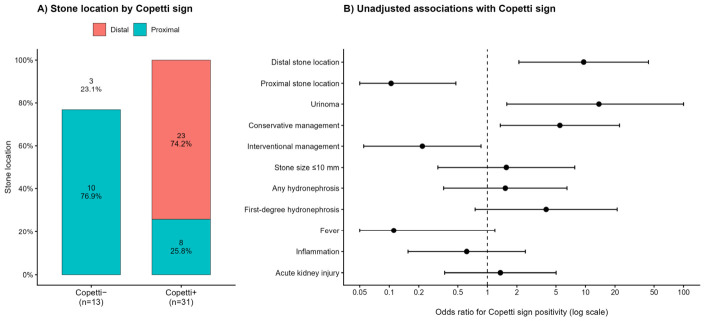
Clinical and sonographic associations of the Copetti sign. (**A**) Distribution of ureteral stone location according to Copetti sign status. Distal ureteral stones were more frequent among Copetti-positive patients than among Copetti-negative patients. (**B**) Forest plot showing unadjusted odds ratios with 95% confidence intervals for variables associated with Copetti sign positivity. The dotted vertical line represents the line of no effect (odds ratio = 1.0). Estimates are exploratory and should be interpreted cautiously given the small sample size and sparse data.

Exploratory analyses of variables associated with Copetti sign positivity are summarized in Figure 2. Distal stone location and urinoma showed the strongest unadjusted associations with Copetti sign positivity. Stone size and management strategy were considered secondary exploratory variables and should be interpreted cautiously given the small sample size and sparse data.

### Diagnostic Performance for Distal Stone Location

The diagnostic performance of the Copetti sign for distal ureteral stone location is reported in Table 2. Sensitivity was 88.5% (95% CI 69.8–97.6%), specificity 55.6% (95% CI 30.8–78.5%), PPV 74.2% (95% CI 55.4–88.1%), and NPV 76.9% (95% CI 46.2–95.0%). The unadjusted odds ratio was 9.58 (95% CI 2.09–43.84; Fisher’s exact *p* = 0.0026).

## 4. Discussion

Our study provides preliminary evidence that the Copetti sign may serve as a promising dynamic sonographic marker for identifying distal ureteral stones in patients with suspected renal colic. Our prospective cohort showed that most patients with the sign had stones that were located distally, supporting and expanding on the original findings by Castelletto et al. [7]. Importantly, our data also revealed an association between the Copetti sign and conservative treatment allocation. This association should not be interpreted as evidence that the Copetti sign causes or guarantees spontaneous passage; rather, it suggests that the sign identifies a clinical-imaging phenotype more often managed non-invasively in our cohort.

The Copetti sign may be related to urine extravasation into Gerota’s fascia due to partial ureteral obstruction, resulting in a thin, undetectable urinoma. This amount of fluid may enable the kidney to freely oscillate within the retroperitoneal space due to pulsatility transmitted by the aorta. This movement may manifest as the Copetti sign on ultrasound [7,8,9]. The higher frequency of urinoma among Copetti-positive patients supports, but does not prove, the proposed mechanism whereby minimal perirenal fluid may facilitate rhythmic kidney oscillation transmitted by aortic pulsatility.

Non-contrast CT remains the diagnostic gold standard for nephrolithiasis due to its high sensitivity and specificity [10]. However, CT is not without limitations, such as radiation exposure, increased cost, and imaging delays [4,11]. Conversely, POCUS provides a rapid, radiation-free bedside assessment, which is particularly beneficial in emergency settings [12,13]. Nonetheless, ultrasound has limited sensitivity for detecting distal stones. Khatiwada et al. reported a sensitivity of 56.5% for distal ureteral stones using ultrasound, compared to 66.7% and 95% for proximal and pelvic-ureteric junction stones, respectively [14]. The Copetti sign may complement traditional sonographic findings by offering a dynamic, motion-based feature associated with distal stone location.

In contrast to Castelletto et al., stone size tended to be smaller among Copetti-positive patients, but this difference did not reach statistical significance in our cohort. Given the small sample size, this finding should be considered exploratory and requires confirmation in larger studies [7].

In our cohort, conservative management was attempted in 77% of Copetti-positive patients, consistent with prior literature supporting non-invasive management for distal stones [15,16]. However, this association should be interpreted as a downstream management association rather than a prognostic effect, because treatment allocation was not standardized and conservative and interventional strategies were not mutually exclusive. Therefore, this finding should be interpreted only as an observed treatment pattern within this single-center cohort, not as evidence of independent prognostic value or validated ability to predict spontaneous stone passage. Future studies should validate this finding using protocolized treatment pathways and patient-centered outcomes.

Our findings reflect a growing trend in emergency ultrasonography toward dynamic diagnostic criteria. Previous work by Wong et al. and Nicolau and Sternberg emphasized the need to move beyond static signs such as hydronephrosis [3,5,13]. The Copetti sign may represent a potentially useful functional addition to the sonographic assessment of renal colic.

Nevertheless, our study has limitations. The small sample size and the limited number of outcome events increase the risk of sparse-data bias and unstable effect estimates. Consequently, the magnitude of the observed odds ratios should be interpreted cautiously, as they may overestimate the strength of the true associations. Furthermore, the available sample size precluded reliable multivariable modelling, and residual confounding cannot be excluded.

No formal assessment of interobserver agreement was performed. Because the Copetti sign is a dynamic sonographic finding requiring operator interpretation, its reproducibility remains uncertain. Future studies should incorporate blinded image review and formal inter-rater reliability testing before broader implementation can be recommended.

The present study was not designed to determine whether the Copetti sign provides incremental diagnostic information beyond established sonographic findings such as hydronephrosis or stone characteristics. Our findings should be validated in wider populations and the integration of the Copetti sign into composite ultrasound-based diagnostic algorithms should be tested in future multicenter studies. Such models may combine dynamic and static sonographic signs to guide clinical decision-making, including imaging requirements, therapeutic strategies, and ED disposition [17].

Although the present study was not designed to evaluate CT avoidance or diagnostic substitution, the Copetti sign could eventually be investigated as part of future POCUS-first pathways. Whether it provides incremental value beyond hydronephrosis, stone size, or established clinical prediction models remains unknown. In this context, the Copetti sign should be viewed as a candidate adjunctive marker for future ultrasound-based algorithms rather than as a standalone replacement for CT or established clinical prediction tools [18,19]. The use of ultrasound-first approaches has been shown to reduce CT utilization, shorten ED stays, and improve patient throughput in previous economic analyses [20,21]. If validated, the Copetti sign could become a useful component of streamlined, patient-centered pathways for nephrolithiasis.

## 5. Conclusions

The Copetti sign may help identify a distal-stone phenotype in patients with suspected renal colic. Its association with conservative management is clinically relevant but requires external validation using patient-centered outcomes such as spontaneous passage, need for delayed intervention, return visits, and pain resolution.

## Figures and Tables

**Table 1:** Baseline clinical, laboratory, and imaging characteristics according to Copetti sign status *.

Variable	All Sample (N = 44)	Copetti Sign Group (N = 31)	Non-Copetti Sign Group (N = 13)	p.Overall
Left flank	21 (47.7%)	14 (45.2%)	7 (53.8%)	0.845
Right flank	23 (52.3%)	17 (54.8%)	6 (46.2%)	0.845
Fever	4 (9.09%)	1 (3.23%)	3 (23.1%)	0.071
Acute Kidney Injury	26 (59.1%)	19 (61.3%)	7 (53.8%)	0.903
Inflammation	27 (61.4%)	18 (58.1%)	9 (69.2%)	0.723
Hydronephrosis absent	9 (20.5%)	6 (19.4%)	3 (23.1%)	1.000
First degree hydronephrosis	15 (34.1%)	13 (41.9%)	2 (15.4%)	0.162
Second degree hydronephrosis	15 (34.1%)	10 (32.3%)	5 (38.5%)	0.737
Third degree hydronephrosis	3 (6.82%)	2 (6.5%)	1 (7.7%)	0.204
Urinoma	17 (39.5%)	16 (53.3%)	1 (7.69%)	0.013
Ureteral jet absent	2 (4.65%)	1 (3.33%)	1 (7.69%)	0.518
Twinkling artifact	3 (6.98%)	3 (10.0%)	0	0.542
Proximal location	18 (40.9%)	8 (25.8%)	10 (76.9%)	0.005
Distal location	26 (59.1%)	23 (74.2%)	3 (23.1%)	0.005
Size (ø mm)	7.5 [4.0–10.0]	6.0 [3.3–8.8]	8 [5.0–10.0]	0.161
Conservative management	29 (65.9%)	24 (77.4%)	5 (38.5%)	0.019
Interventional management	16 (36.4%)	8 (25.8%)	8 (61.5%)	0.040

* Categorical variables are described as absolute frequencies (and percentages); continuous variables are described as median (and interquartile range). Percentages were calculated using available non-missing data for each variable.

**Table 2:** Diagnostic performance of the Copetti sign for distal ureteral stone location. Sensitivity, specificity, positive predictive value (PPV), negative predictive value (NPV), unadjusted odds ratio (OR), and Fisher’s exact *p*-value are reported.

Metric	Value (95% CI)
Sensitivity	88.5% (69.8–97.6%)
Specificity	55.6% (30.8–78.5%)
PPV	74.2% (55.4–88.1%)
NPV	76.9% (46.2–95.0%)
OR	9.58 (95% CI 2.09–43.84)
Fisher *p*	0.0026

## Data Availability

The data supporting the findings of this study are available from the corresponding author upon reasonable request.

## Supplementary Materials

The following supporting information can be downloaded at: https://www.mdpi.com/article/10.3390/healthcare14121663/s1, Video S1: Dynamic ultrasound appearance of the Copetti sign.

## Abbreviations

The following abbreviations are used in this manuscript:

BP	Blood pressure
CT	Computed tomography
ED	Emergency department
IRB	Institutional Review Board
POCUS	Point-of-care ultrasound
NPV	Negative predictive value
OR	Odds ratio
PPV	Positive predictive value
CI	Confidence interval
IQR	Interquartile range
MET	Medical expulsive therapy

## References

[1] Cupisti A., Pasquali E., Lusso S., Carlino F., Orsitto E., Melandri R. (2008). Renal colic in Pisa emergency department: Epidemiology, diagnostics and treatment patterns. Intern. Emerg. Med..

[2] Smith-Bindman R., Aubin C., Bailitz J., Bengiamin R.N., Camargo C.A., Corbo J., Dean A.J., Goldstein R.B., Griffey R.T., Jay G.D. (2014). Ultrasonography versus computed tomography for suspected nephrolithiasis. N. Engl. J. Med..

[3] Nicolau C., Claudon M., Derchi L.E., Adam E.J., Nielsen M.B., Mostbeck G., Owens C.M., Nyhsen C., Yarmenitis S. (2015). Imaging patients with renal colic—Consider ultrasound first. Insights Imaging.

[4] Schoenfeld E.M., Pekow P.S., Shieh M.S., Scales C.D., Lagu T., Lindenauer P.K. (2017). The diagnosis and management of patients with renal colic across a sample of US hospitals: High CT utilization despite low rates of admission and inpatient urologic intervention. PLoS ONE.

[5] Wong C., Teitge B., Ross M., Young P., Robertson H.L., Lang E. (2018). The accuracy and prognostic value of point-of-care ultrasound for nephrolithiasis in the emergency department: A systematic review and meta-analysis. Acad. Emerg. Med..

[6] Ray A.A., Ghiculete D., Pace K.T., Honey R.J. (2010). Limitations to ultrasound in the detection and measurement of urinary tract calculi. Urology.

[7] Castelletto S., Amore G., Giudice C.A., Orso D., Copetti R. (2022). A preliminary investigation on the “swinging kidney”: A sonographic sign useful for diagnosing renal colic. J. Diagn. Med. Sonogr..

[8] Öğreden E., Oǧuz U., Karadayı M., Demirelli E., Tosun A., Günaydın M. (2019). Factors associated with urinoma accompanied by ureteral calculi. Arch. Ital. Urol. Androl..

[9] Thom C., Eisenstat M., Moak J. (2018). Point-of-care ultrasound identifies urinoma complicating simple renal colic: A case series and literature review. J. Emerg. Med..

[10] Ripollés T., Agramunt M., Errando J., Martínez M.J., Coronel B., Morales M. (2004). Suspected ureteral colic: Plain film and sonography vs unenhanced helical CT. A prospective study in 66 patients. Eur. Radiol..

[11] Malaki M. (2014). The comparison of ultrasound and non-contrast helical computerized tomography for children nephrolithiasis detection. Urol. Ann..

[12] Pathan S.A., Mitra B., Mirza S., Momin U., Ahmed Z., Andraous L.G., Shukla D., Shariff M.Y., Makki M.M., George T.T. (2018). Emergency physician interpretation of point-of-care ultrasound for identifying and grading of hydronephrosis in renal colic compared with consensus interpretation by emergency radiologists. Acad. Emerg. Med..

[13] Sternberg K.M., Pais V.M., Larson T., Han J., Hernandez N., Eisner B. (2016). Is hydronephrosis on ultrasound predictive of ureterolithiasis in patients with renal colic?. J. Urol..

[14] Khatiwada B., Mahat A., Yadav G.K., Duwadi B., Mishra U., Bhusal A., Yadav P., Khadka H. (2024). A comparative study of ultrasonography (USG) and computed tomography for detecting ureteric calculi in patients with acute flank pain, and analysis of factors influencing ultrasound detection rates. Int. J. Surg. Glob. Health.

[15] Orso D., Peric D., Di Gioia C.C., Comisso I., Bove T., Ban A., Fonda F., Federici N. (2024). Renal and genitourinary ultrasound evaluation in emergency and critical care: An overview. Healthcare.

[16] Jendeberg J., Geijer H., Alshamari M., Cierzniak B., Lidén M. (2017). Size matters: The width and location of a ureteral stone accurately predict the chance of spontaneous passage. Eur. Radiol..

[17] Schoenfeld E.M., Poronsky K.E., Westafer L.M., DiFronzo B.M., Visintainer P., Scales C.D., Hess E.P., Lindenauer P.K. (2021). Feasibility and efficacy of a decision aid for emergency department patients with suspected ureterolithiasis: Protocol for an adaptive randomized controlled trial. Trials.

[18] Tung Chen Y., Rodríguez Fuertes P., Oliver Sáez P., Villegas V., Soto B., Calle F., Cardona C., Mora C., Cañadas J., Tong Y. (2021). Efficacy of a fast-track pathway for managing uncomplicated renal or ureteral colic in a hospital emergency department: The STONE randomized clinical trial of Sonography and Testing of a Nephrolithiasis Episode. Emergencias.

[19] Daniels B., Gross C.P., Molinaro A., Singh D., Luty S., Jessey R., Moore C.L. (2016). STONE PLUS: Evaluation of emergency department patients with suspected renal colic, using a clinical prediction tool combined with point-of-care limited ultrasonography. Ann. Emerg. Med..

[20] Faget C., Millet I., Sebbane M., Thuret R., Verheyden C., Curros-Doyon F., Molinari N., Taourel P. (2021). Imaging strategies for patients with suspicion of uncomplicated colic pain: Diagnostic accuracy and management assessment. Eur. Radiol..

[21] Doty E., DiGiacomo S., Gunn B., Westafer L., Schoenfeld E. (2021). What are the clinical effects of the different emergency department imaging options for suspected renal colic? A scoping review. J. Am. Coll. Emerg. Physicians Open.

